# Impact of GnRH antagonist protocols versus progestin-primed ovarian stimulation on reproductive outcomes in advanced reproductive age women: a propensity score-matched retrospective cohort study

**DOI:** 10.3389/fendo.2026.1781506

**Published:** 2026-05-14

**Authors:** Zhaokang Qi, Tingting Wang, Jinxin Ren, Shuai Zhao, Jingyan Song, Fang Lian, Yuewen Zhao

**Affiliations:** 1 The First Clinical Medical College, Shandong University of Traditional Chinese Medicine, Jinan, China; 2 Department of Reproduction and Genetics, Affiliated Hospital of Shandong University of Traditional Chinese Medicine, Jinan, China; 3 CReATe Fertility Centre, Toronto, ON, Canada

**Keywords:** ARA women, GnRH antagonist protocol, infertility, live birth rate, PPOS protocol

## Abstract

**Purpose:**

This study aims to compare the live birth rate (LBR) and reproductive outcomes between gonadotropin-releasing hormone antagonist (GnRH-ant) and progestin-primed ovarian stimulation (PPOS) protocols in infertile women of advanced reproductive age (ARA).

**Methods:**

A retrospective analysis was conducted on 1588 female patients with ARA who underwent *in vitro* fertilization or intracytoplasmic sperm injection at the Reproductive and Genetic Center of the Affiliated Hospital of Shandong University of Traditional Chinese Medicine between January 1, 2020, and February 29, 2024, and met the inclusion and exclusion criteria. To ensure scientific rigor, propensity score matching (1:1) was applied to balance the baseline characteristics. Ultimately, reproductive outcomes were analyzed for GnRH-ant (n = 277) and PPOS (n = 277) protocol groups.

**Results:**

Non-significant differences were observed in baseline characteristics between the two groups. Regarding cycle data, the total gonadotropin dosage was significantly lower in the GnRH-ant group (*P* < 0.001), whereas the duration of gonadotropin stimulation was slightly longer (*P =* 0.004). On the trigger day, serum luteinizing hormone (*P* = 0.002), estradiol (*P* = 0.038), and progesterone (*P* < 0.001) levels were significantly higher in the GnRH-ant group compared to the PPOS group. Regarding oocyte retrieval and embryo outcomes, the GnRH-ant group demonstrated significantly higher numbers of oocytes retrieved (*P* < 0.001), 2-pronuclei fertilized oocytes (*P* = 0.003), cleavage-stage embryos (*P* < 0.001), and blastocysts (*P =* 0.010), indicating superior embryo development outcomes compared to the PPOS group. For pregnancy outcomes, 159 and 262 transfer cycles were performed in the GnRH-ant and PPOS groups, respectively. The GnRH-ant group exhibited significantly higher rates than the PPOS group in clinical pregnancy rate (CPR) (*P* = 0.024), ongoing pregnancy rate (*P* = 0.018), and LBR (*P* = 0.045), reflecting favorable cycle outcomes. Although the GnRH-ant group had a higher implantation rate (*P* = 0.066), these differences did not reach statistical significance. The PPOS group exhibited a lower miscarriage rate compared to the GnRH-ant group; however, this difference was also statistically non-significant (*P* = 0.981). Statistically non-significant difference was observed in ectopic pregnancy rates between the groups, and no cases of ovarian hyperstimulation syndrome occurred in either group. Finally, multivariate logistic regression analysis revealed that the treatment protocol was an independent factor influencing the LBR. The probability of live birth in the GnRH-ant group was significantly higher than that in the PPOS group (odds ratio = 6.085, 95% confidence interval: 2.489–14.880, *P* < 0.001).

**Conclusion:**

This study indicates that the GnRH-ant stimulation protocol is advantageous as compared to the PPOS protocol for infertile women with ARA undergoing controlled ovarian stimulation combined with frozen embryo transfer, as evidenced by improved embryological outcomes, higher CPRs, and higher LBRs.

## 1 Introduction

Epidemiological studies indicate that the global prevalence of infertility among individuals aged 15–49 years significantly increased between 1990 and 2021, and this trend is projected to persist until 2040 (1, 2). Concurrently, the infertility rate in China has increased from 3% to approximately 12.5%–15% over the past two decades. Increased access to higher education and career development opportunities for modern women has led more to postpone childbearing. Women over 35 years exhibit a higher incidence of infertility, which severely impacts their physical and mental health (3–5). Against this backdrop, assisted reproductive technology (ART) has become increasingly vital for infertility treatment. Selecting the optimal *in vitro* fertilization and embryo transfer (*in vitro* fertilization (IVF)-ET) treatment strategy is crucial for enhancing success rates and reducing costs (6).

Controlled ovarian stimulation (COS), a critical step in ART, aims to overcome the quantitative limitations of natural ovulation using exogenous gonadotropins (Gn) to induce multifollicular development and obtain multiple embryos (7). Under physiological conditions, the progressively rising estradiol (E2) levels during the follicular phase shift from inhibiting to stimulating Gn-releasing hormone (GnRH) release, subsequently triggering GnRH surge and its downstream luteinizing hormone (LH) surge (8, 9). However, retrospective studies suggest that a premature LH surge can induce early ovulation, compromise oocyte quality, and reduce the live birth rate (LBR) (10). Therefore, effectively suppressing premature LH surges is a key factor in optimizing COS outcomes. Commonly used clinical protocols to control premature LH surges include GnRH antagonist and progestin-primed ovarian stimulation (PPOS) protocols.

During COS, GnRH agonists or GnRH antagonists are widely used for pituitary downregulation to suppress the endogenous LH surge (11). The GnRH agonist protocol uses an initial “flare-up” effect to transiently stimulate the pituitary gland to release large amounts of follicle-stimulating hormone (FSH) and LH, promoting synchronized follicular development (12). However, prolonged exposure to GnRH agonists leads to a reduction in GnRH receptor numbers, thereby inhibiting LH release (13). Conversely, the GnRH antagonist (GnRH-ant) protocol directly and rapidly blocks GnRH receptors, effectively preventing a premature LH surge (14). This avoids abnormal follicular development, decreased oocyte quality, or premature ovulation caused by an early LH surge. A large-sample meta-analysis indicates that, compared to the GnRH agonist protocol, the GnRH-ant protocol significantly reduces the risk of ovarian hyperstimulation syndrome (OHSS) while maintaining comparable LBR and ongoing pregnancy rate (15). Given these efficacy and safety advantages, the GnRH antagonist protocol has become an increasingly preferred COS regimen.

Using exogenous progestins to replace GnRH analogues for pituitary downregulation has emerged as an effective COS method (16). When persistently elevated estrogen levels reach a threshold during the late follicular phase, they exert a positive feedback effect, stimulating the hypothalamic arcuate nucleus to release numerous GnRH. This subsequently induces a pituitary LH surge, leading to ovulation (8). PPOS, an ovarian stimulation protocol based on a freeze-all strategy, was first proposed in 2015. It leverages the anti-estrogenic properties of progestins to effectively suppress premature LH surges triggered by rising estrogen levels during the follicular phase (16–18). Compared to GnRH analogue protocols, the PPOS protocol provides significant advantages regarding convenient oral administration and lower medication costs (16). Medroxyprogesterone acetate (MPA) has been successfully used as an adjuvant to Gn in the PPOS protocol (19).

Existing research suggests that PPOS yields cumulative LBR comparable to the GnRH-ant protocol in young IVF/intracytoplasmic sperm injection (ICSI) women with low anti-Müllerian hormone (AMH) levels (< 0.5 ng/mL) (20). A study by Li J et al. (21) involving 89 women with diminished ovarian reserve following laparoscopic excision of ovarian endometriotic cysts compared pregnancy outcomes between GnRH-ant protocol and PPOS. The results revealed that while the clinical pregnancy rate and cumulative LBR were numerically higher in the GnRH-ant protocol group, the differences between the two groups were statistically non-significant. However, this study did not include infertile women aged > 35 years. Consequently, well-designed studies comparing the efficacy of the GnRH-ant protocol versus the PPOS protocol specifically in ARA infertile women are urgently required.

This retrospective study aimed to compare the reproductive outcomes of the GnRH-ant and the PPOS protocols in IVF/ICSI cycles of ARA infertile women. This study aimed to provide evidence for selecting a more suitable ovarian stimulation protocol for infertile women aged ≥ 35 years.

## 2 Materials and methods

### 2.1 Study design

**Figure 1:**
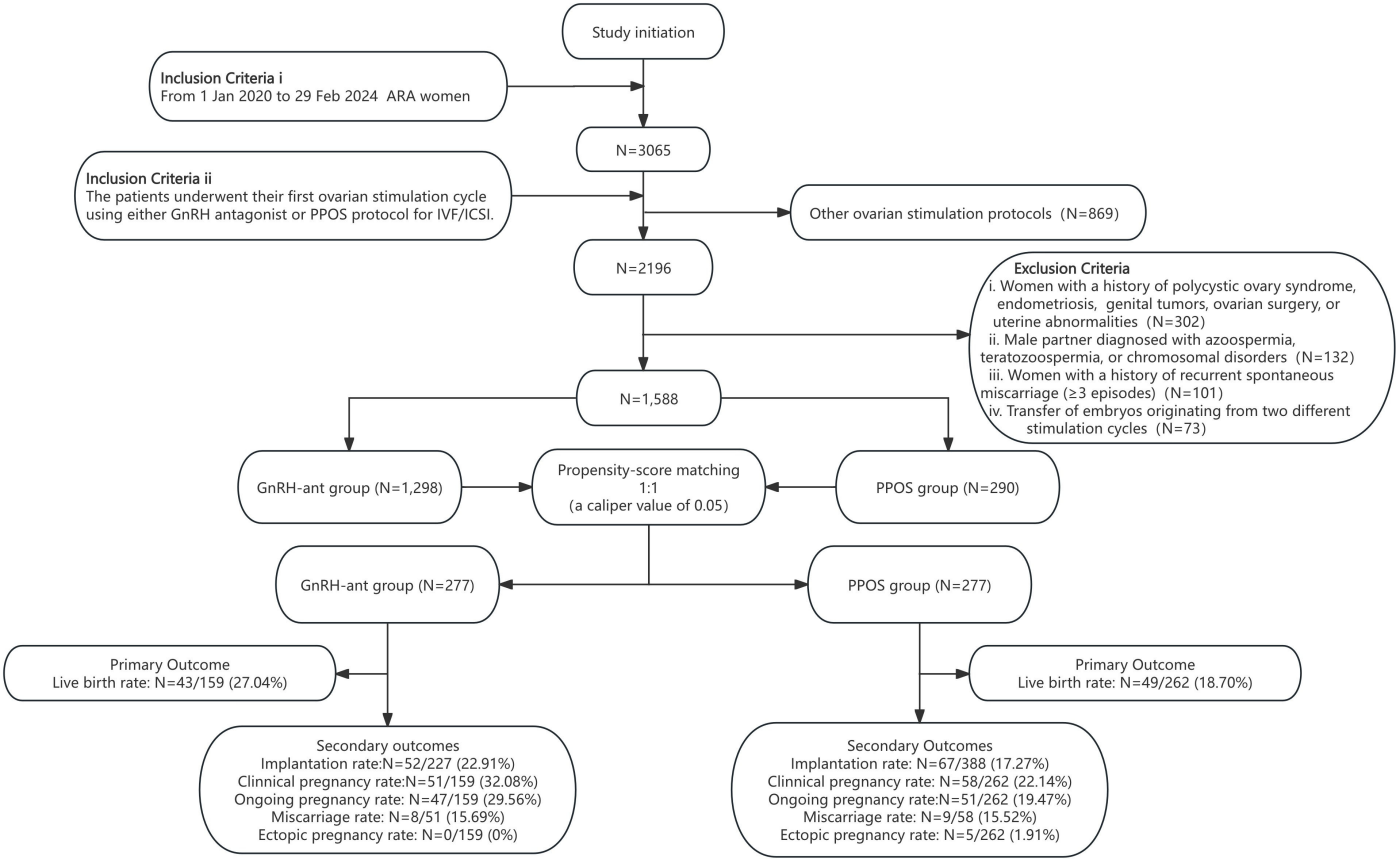
Study flowchart.

This retrospective study analyzed data from infertile women who underwent their first IVF or ICSI treatment cycle at our center between January 1, 2020, and February 29, 2024. The study protocol was approved by the Reproductive Medicine Ethics Committee of the Affiliated Hospital of Shandong University of Traditional Chinese Medicine [Approval Number: 2025-079-02-KY]. As this study used a retrospective cohort design utilizing anonymized historical data, it met the criteria for a waiver of informed consent, as stipulated by the Ethics Committee (Study Flowchart, **Figure 1**).

### 2.2 Inclusion and exclusion criteria

#### 2.2.1 Inclusion criteria

- ARA women ≥ 35 years;
- The patients underwent their first ovarian stimulation cycle using either GnRH-ant or PPOS protocol for IVF/ICSI.

#### 2.2.2 Exclusion criteria

- Women with a history of polycystic ovary syndrome, endometriosis, genital tumors, ovarian surgery, or uterine abnormalities;
- Male partner diagnosed with azoospermia, teratozoospermia, or chromosomal disorders;
- Women with a history of recurrent spontaneous miscarriage (≥ 3 episodes);
- Transfer of embryos originating from two different stimulation cycles.

Most data were extracted from the patients’ electronic medical records of their IVF/ICSI cycles. To supplement the missing information, telephone follow-ups were conducted on the day of oocyte retrieval, and hospital records were reviewed. Based on inclusion and exclusion criteria, 1588 IVF/ICSI cycles were initially enrolled. Cycles from the two groups were matched for cycle characteristics using standard propensity score matching (PSM). Ultimately, 554 IVF/ICSI cycles were included and divided into two groups: GnRH-ant (n = 277) and PPOS (n = 277) protocols.

### 2.3 Ovarian stimulation protocols

#### 2.3.1 GnRH antagonist protocol

Transvaginal ultrasound and baseline hormone assessments were performed on menstrual cycle days 2–4. In the absence of dominant follicles or functional ovarian cysts, recombinant human follicle-stimulating hormone alfa (r-hFSH-α; Gonal-f, Merck Serono GmbH, Switzerland) was administered subcutaneously at a dose of 150–300 IU/d, starting on stimulation days 2–4 and continuing daily until the trigger day. Follicular development was monitored via transvaginal ultrasound on stimulation day 5. When at least one follicle reached ≥ 12 mm in mean diameter, cetrorelix acetate (Cetrotide, Merck Serono LLC, USA) was added at 0.25 mg/day via subcutaneous injection, administered daily until the trigger day.

#### 2.3.2 PPOS protocol

On menstrual cycle days 2–4, transvaginal ultrasound and baseline hormone assessments were performed. MPA (Zhejiang Xianju Pharmaceutical Co., Ltd., China) 4 mg twice daily (BID) was initiated orally in combination with human menopausal gonadotropin (HMG; Livzon Pharmaceutical Group Inc., China) 150–300 IU/day administered subcutaneously daily until the trigger day. Follicular development was monitored via serial transvaginal ultrasound every 2–3 days, and the HMG dose was adjusted based on the follicular diameter and serum estradiol levels.

### 2.4 Embryo transfer and endometrial preparation protocols

#### 2.4.1 Embryo transfer strategy

For patients who underwent either GnRH-ant or PPOS protocol and were classified as using the frozen embryo transfer (FET) strategy, luteal phase support (LPS) was initiated on the day of embryo thawing and continued until 10 weeks of gestation. The LPS regimen was as follows: Progesterone in oil (Zhejiang Xianju Pharmaceutical Co., Ltd., China) 40 mg intramuscularly once daily, and dydrogesterone (Duphaston, Abbott B.V., Netherlands) 10 mg orally twice daily.

#### 2.4.2 Endometrial preparation protocol

Endometrial preparation for FET cycles used a step-up hormone replacement therapy protocol: Oral estradiol valerate (Progynova, Zhejiang Xianju Pharmaceutical Co., Ltd., China) 4 mg/day was initiated on menstrual cycle days 2–4. The dose was escalated every 4 days: 6 mg/day on days 6–9 and 8 mg/day on days 10–13. From day 14 onward, the dose was adjusted based on serial transvaginal ultrasound (endometrial thickness) and serum estradiol levels. Once the endometrial thickness reached ≥ 8 mm, progesterone conversion was initiated. Starting on the day of progesterone conversion (designated P + 0), LPS comprised progesterone in oil (40 mg intramuscularly once daily). Duphaston 10 mg orally BID. Embryo transfer was scheduled after progesterone initiation according to the embryo developmental stage, with cleavage-stage embryos (D3) transferred on P + 4 or blastocysts (D5) transferred on P + 6. Serum β-human chorionic gonadotropin (β-hCG) was measured 14 days post-ET. A level ≥ 60 mIU/mL defined biochemical pregnancy, with LPS continued until 10 gestational weeks. Embryo transfer was typically scheduled for either cleavage-stage embryos (D3) or blastocysts (D5) after progesterone initiation.

### 2.5 Outcome measures

#### 2.5.1 Baseline characteristics

The following baseline data were recorded for patients in both groups: Age, gravidity, parity, body mass index (BMI), AMH, antral follicle count (AFC), basal FSH, and basal LH.

#### 2.5.2 Laboratory and cycle data

The following laboratory and cycle outcome measures were analyzed: Duration of Gn, Total Gn, LH level on trigger day, E2 level on trigger day, P level on trigger day, number of oocytes retrieved, number of 2-pronuclei (2PN), number of cleavage-stage embryos, and number of blastocysts formed. Cleavage-stage embryos were evaluated and scored using morphological criteria (22). Blastocysts were assessed and graded using the Gardner and Schoolcraft classification system (23).

#### 2.5.3 Pregnancy outcome data

##### 2.5.3.1 Primary outcome measure

The primary outcome measure was the LBR, which was defined as follows:

LBR: The proportion of transfer cycles resulting in the delivery of at least one live-born infant among all transfer cycles. A live birth is defined as the complete expulsion or extraction of a product of conception from its mother that, after separation, breathes or exhibits any other evidence of life.

##### 2.5.3.2 Secondary outcome measures

Secondary outcome measures included implantation rate, clinical Pregnancy Rate, ongoing pregnancy rate, miscarriage rate, ectopic pregnancy rate, and OHSS. These were defined as follows:

- Implantation Rate: (Number of gestational sacs observed)/(Number of embryos transferred) × 100%.
- Clinical Pregnancy: Visualization of an intrauterine gestational sac by ultrasound, occurring 14 days after a positive blood/urine pregnancy test.
- Clinical Pregnancy Rate: The proportion of transfer cycles resulting in a clinical pregnancy among all transfer cycles.
- Ongoing Pregnancy Rate: The proportion of transfer cycles resulting in a clinical pregnancy that progressed beyond 12 weeks of gestation with fetal cardiac activity confirmed by ultrasound among all transfer cycles.
- Miscarriage: Spontaneous loss of a clinical pregnancy before 28 weeks of gestation, with a fetal weight of < 1000 g.
- Miscarriage Rate: The proportion of clinical pregnancies that ended in miscarriage.
- Ectopic Pregnancy Rate: The proportion of transfer cycles resulting in an ectopic pregnancy among all transfer cycles.
- OHSS Incidence: The proportion of oocyte retrieval cycles complicated by OHSS among all oocyte retrieval cycles.

### 2.6 Data management

Two researchers independently screened ARA infertile women treated at the Reproductive and Genetic Center of the Affiliated Hospital of Shandong University of Traditional Chinese Medicine who met the inclusion criteria using ART. Patient baseline characteristics and pregnancy outcome data were extracted and entered into an Excel spreadsheet. Subject selection strictly adhered to the predefined inclusion and exclusion criteria. To ensure data accuracy, a dual-entry verification process was implemented: Data were entered twice by two different individuals and subsequently cross-checked item by item. Any discrepancies identified during this verification process were resolved through re-extraction of the data from the source records or additional follow-up, followed by corrected data entry.

### 2.7 Statistical analysis

To ensure scientific rigor, statistical analyses were performed using the Statistical Package for the Social Sciences software (version 30.0) and R programming language. The sample size calculation was based on the primary outcome measure, LBR. Assuming an anticipated difference of 15% between the two groups, with a significance level (α) of 0.05 and a statistical power of 80%, a minimum of 190 participants were required per group. Accounting for a potential dropout rate of 20%, the final sample size was adjusted to include at least 238 participants per group, with as many eligible cases as possible included in the actual study. Standard PSM with nearest neighbor matching and a caliper value of 0.05 was applied to achieve 1:1 matching based on the following baseline characteristics: Age, gravidity, parity, BMI, AMH, AFC, basal FSH, and basal LH. Unmatched cases were excluded from the analysis. After matching, the standardized mean difference (SMD) was used to assess the balance of each covariate between the two groups. An absolute SMD value of < 0.1 is generally considered to indicate a clinically non-significant difference between the groups and an acceptable balance. Intergroup comparisons were performed using the Student’s t-test, Mann–Whitney U test, or χ^2^ test, as appropriate. Continuous variables that met the assumptions of homogeneity of variance and normal distribution were compared using independent samples t-tests. Results are presented as mean ± standard deviation. Non-normally distributed continuous data are described using the median (interquartile range) and compared using the Mann–Whitney U test. For normally distributed data involving more than two groups, a one-way analysis of variance was performed. Categorical variables are presented as frequency (percentage) and compared using the χ^2^ test or Fisher’s exact test, as appropriate. Results are presented as the mean with its 95% confidence interval (CI).

Multivariable Analysis: To control potential confounding factors, a multivariable logistic regression model was used to identify independent factors influencing the LBR. The scheme type was treated as a categorical variable. The variables included in the model were: Age, gravidity, parity, BMI, AMH, AFC. Statistical significance was defined as a two-sided *P*-value < 0.05.

## 3 Results

Among the 1588 women who met the inclusion and exclusion criteria, 1298 were treated using the GnRH-ant protocol, and 290 were treated using the PPOS protocol. To minimize bias arising from imbalanced baseline characteristics, PSM was performed using a 1:1 matching ratio. Matching was based on the following variables: Female age, gravidity, parity, BMI, AMH, AFC, basal FSH, and basal LH. After matching, 277 matched pairs were retained in each group. The baseline demographic and clinical characteristics of the matched cohorts are presented in **Table 1**. All baseline characteristics exhibited non-significant differences between the two groups (*P* > 0.05), indicating that the groups were comparable.

**Table 1:** Baseline characteristics of patients.

Variables	GnRH-ant group (N = 277)	PPOS group (N = 277)	P-value	SMD
Age (years)	40.21 (36.64-43.78)	40.25 (36.64-43.86)	0.906	-0.011
Gravidity	1.73 (0.23-3.23)	1.65 (0.17-3.13)	0.531	0.054
Parity	1.00(0.00-1.00)	0.00(0.00-1.00)	0.290	0.027
BMI (kg/m²)	23.94 (20.70-27.18)	23.87 (20.39-27.35)	0.795	0.021
AMH (ng/mL)	0.90 (0.29-1.59)	0.81 (0.18-1.44)	0.080	0.141
AFC	7.64 (3.28-12.00)	7.10 (3.49-10.71)	0.114	0.135
Basal FSH(mIU/mL)	8.65 (5.41-11.89)	8.58 (4.89-12.27)	0.797	0.020
Basal LH (mIU/mL)	4.38 (2.23-6.53)	4.22 (1.75-6.69)	0.452	0.069

### 3.1 Baseline characteristics of patients

No substantial variations existed between the two groups regarding age (40.21 versus 40.25 years), gravidity (1.73 versus 1.65), parity (1.00 versus 0.00), BMI (23.94 versus 23.87 kg/m^2^), AMH (0.90 versus 0.81 ng/mL), AFC (7.64 versus 7.10), basal FSH (8.65 versus 8.58 mIU/mL), and basal LH (4.38 versus 4.22 mIU/mL) (**Table 1**).

### 3.2 Cycle parameters

Compared to the PPOS protocol group, the GnRH-ant required a significantly lower total Gn dosage (2573.29 IU versus 3541.59 IU, *P* < 0.001); however, it had a slightly longer duration of stimulation (9.00 days versus 8.53 days, *P* = 0.004). On the trigger day, the GnRH-ant protocol group exhibited significantly higher serum levels of LH (3.83 mIU/mL versus 3.18 mIU/mL, *P* = 0.002), E2 (1260.60 pg/mL versus 1102.33 pg/mL, *P* = 0.038), and P (0.83 ng/mL versus 0.69 ng/mL, *P* < 0.001) (**Table 2**).

**Table 2:** Cycle parameters.

Variables	GnRH-ant group (N = 277)	PPOS group (N = 277)	P-value
Total Gn Dosage (IU)	2573.29 (1513.23-3633.35)	3541.59 (2403.06-4680.12)	< 0.001
Duration of Stimulation (days)	9.00 (7.10-10.90)	8.53 (6.65-10.41)	0.004
Trigger Day LH (mIU/mL)	3.83 (1.23-6.43)	3.18 (0.82-5.54)	0.002
Trigger Day E2(pg/mL)	1260.60 (348.71-2172.49)	1102.33 (220.26-1984.40)	0.038
Trigger Day P (ng/mL)	0.83 (0.35-1.31)	0.69 (0.30-1.08)	< 0.001

### 3.3 Oocyte and embryo outcomes

Compared to the PPOS protocol group, the GnRH-ant protocol group demonstrated significantly higher values for the number of oocytes retrieved (4.51 versus 3.66, *P* < 0.001), number of 2PN zygotes (3.13 versus 2.64, *P* = 0.003), number of Day 3 embryos (1.41 versus 1.07, *P* < 0.001), and number of Day 5 embryos (1.11 versus 0.92, *P =* 0.010), indicating superior embryological outcomes with the GnRH-ant protocol (**Table 3**).

**Table 3:** Oocyte and embryo outcomes.

Variables	GnRH-ant group (N = 277)	PPOS group (N = 277)	P-value
Number of Oocytes Retrieved	4.51 (1.40-7.61)	3.66 (1.39-5.93)	< 0.001
Number of 2PN Zygotes	3.13 (1.00-5.26)	2.64 (0.89-4.39)	0.003
Number of Day 3 Embryos	1.41 (0.15-2.67)	1.07 (0.28-1.86)	< 0.001
Number of Day 5 Embryos	1.11 (0.22-2.00)	0.92 (0.06-1.78)	0.010

### 3.4 Pregnancy outcomes in FET Cycles

In this study, only frozen-thawed embryo transfer cycles were included in both groups, with 159 cycles in the GnRH-ant protocol group and 262 cycles in the PPOS protocol group. A total of 227 embryos were transferred to the GnRH group, whereas 388 embryos were transferred to the PPOS group. The GnRH-ant protocol group demonstrated significantly higher clinical pregnancy rate (32.08% versus 22.14%, *P* = 0.024), ongoing pregnancy rate (29.56% versus 19.47%, *P* = 0.018), and LBR (27.04% versus 18.70%, *P* = 0.045) compared with the PPOS protocol group. Although the implantation rate was numerically higher in the GnRH-ant protocol group (22.91% versus 17.27%), the difference did not reach statistical significance (*P* = 0.066). Non-significant differences were observed between the two groups regarding miscarriage rate (15.69% versus 15.52%, *P* = 0.981) or ectopic pregnancy rate (0% versus 1.91%, *P* = 0.162). No cases of ovarian hyperstimulation syndrome were observed in either group (**Table 4**).

**Table 4:** Pregnancy outcomes in FET cycles.

Variables	GnRH-ant group (N = 277)	PPOS group (N = 277)	OR (95% CI)	P-value
Transfer Cycles	N=159	N=262	/	/
Number of Embryos Transferred	N=227	N=388	/	/
Implantation Rate (%)	52/227 (22.91%)	67/388 (17.27%)	1.517(0.971-2.369)	0.066
Clinical Pregnancy Rate (%)	51/159 (32.08%)	58/262 (22.14%)	1.661(1.067-2.586)	0.024
Ongoing Pregnancy Rate (%)	47/159 (29.56%)	51/262 (19.47%)	1.736(1.098-2.744)	0.018
Live Birth Rate (%)	43/159 (27.04%)	49/262 (18.70%)	1.611(1.009-2.573)	0.045
Miscarriage Rate (%)	8/51 (15.69%)	9/58 (15.52%)	1.062(0.376-3.002)	0.981
Ectopic Pregnancy Rate (%)	0/159 (0%)	5/262 (1.91%)	1.019(1.002-1.037)	0.162

### 3.5 Regression analysis

In this study, multivariate logistic regression analysis, after adjusting for confounding factors including age, gravidity, parity, BMI, AMH, AFC, identified the treatment protocol as an independent factor influencing the LBR. The probability of live birth in the GnRH-ant group was significantly higher than that in the PPOS group (odds ratio [OR] = 6.085, 95% CI: 2.489–14.880, *P* < 0.001). Conversely, age, gravidity, parity, BMI, AMH, AFC exhibited no independent association with LBR (all *P* > 0.05) (**Table 5**).

**Table 5:** Multivariate logistic regression analysis of factors associated with live birth rate.

Variables	β	Standard error	Waldχ²	P-Value	OR (95% CI)
Treatment protocol: GnRH-ant protocol	1.806	0.456	15.668	< 0.001	6.085(2.489-14.880)
Age (years)	0.044	0.067	0.421	0.516	1.045 (0.915-1.192)
Gravidity	-0.192	0.222	0.743	0.389	0.826 (0.534-1.276)
Parity	0.121	0.399	0.092	0.762	1.128 (0.517-2.465)
BMI (kg/m²)	-0.009	0.052	0.029	0.865	0.991 (0.894-1.098)
AMH (ng/mL)	-0.179	0.244	0.54	0.462	0.836 (0.518-1.349)
AFC	0.024	0.046	0.283	0.595	1.025 (0.937-1.121)

## 4 Discussion

This study represents the first retrospective analysis comparing reproductive outcomes between GnRH-ant and PPOS protocols in infertile women aged ≥ 35 years undergoing their first ovarian stimulation cycle, thereby addressing a critical evidence gap for personalized protocol selection in this population. Our findings demonstrate that the GnRH-ant stimulation protocol is advantageous as compared to the PPOS protocol for infertile women with ARA undergoing controlled ovarian stimulation combined with frozen embryo transfer, as evidenced by improved embryological outcomes, higher CPRs, and higher LBRs.

Previous studies have demonstrated that FSH and LH secreted by the pituitary gland stimulate the ovaries to synthesize E2 and P, which regulate endometrial receptivity, a critical factor influencing embryo implantation (24). Regarding the mechanisms of controlled ovarian stimulation, there are fundamental differences between the two protocols regarding pituitary inhibition. The GnRH-ant protocol exerts its effect through competitive binding of GnRH-ants to pituitary GnRH receptors, reversibly suppressing premature LH surges and preventing premature ovulation. Pituitary function rapidly recovers upon discontinuation of the antagonist. Conversely, the PPOS protocol uses exogenous progesterone to induce negative feedback on the hypothalamic-pituitary axis. This results in the sustained suppression of pituitary function throughout the stimulation cycle, thereby preventing a premature LH surge (25). Moreover, premature elevation of progesterone levels in the PPOS protocol accelerates the histological maturation of the endometrium, leading to a shift in the implantation window (26). Given that elevated progesterone levels on the day of hCG administration impair endometrial receptivity and reduce pregnancy maintenance rates, the PPOS protocol is typically not followed by a fresh embryo transfer (27). Consequently, to eliminate the confounding effect of endometrial receptivity on pregnancy outcomes, this study uniformly adopted a freeze-thaw embryo transfer strategy.

This study demonstrated that the GnRH-ant protocol exhibits superior ovarian stimulation characteristics in women with ARA, manifested by a significantly reduced total Gn dosage coupled with a moderately extended stimulation duration. These observations suggest that the protocol may optimize diminished ovarian responsiveness in this population through enhanced follicular synchronization recruitment and physiological regulation of the LH window (28). Furthermore, the GnRH-ant protocol group yielded a significantly higher number of blastocysts, consistent with prior reports of improved embryo quality and euploidy rates (29, 30). This indicates potential protective or enhancing effects on oocyte competence and early embryonic development. For patients with ARA and severely limited embryo resources, generating more high-quality blastocysts directly expands the selection pool for embryo transfer, representing a substantial clinical advantage. For women with ARA, AMH levels are predictive of ovarian reserve and embryonic outcomes (31). Although AMH levels between the two groups were not significantly different, the slightly higher AMH levels observed in the GnRH-ant group may partially explain the improved embryological outcomes in this group.

Current guidelines and expert consensus recognize LBR as the gold standard for pregnancy outcomes in assisted reproductive technology (32, 33). This study identified that among women with ARA, the LBR was significantly higher with the GnRH-ant protocol as compared to the PPOS protocol. Previous studies have observed that there was non-significant difference in LBR between GnRH-ant and the PPOS protocols (34–36). The pregnancy outcomes in this study were strongly correlated with embryology laboratory data. The relatively poorer CPR, ongoing pregnancy rate, and LBR observed in the PPOS protocol group likely stem from the inadequate quality and developmental potential of the embryos obtained from the stimulation. The underlying mechanism may be associated with the potential negative impact of a sustained high progesterone environment inherent to the PPOS protocol. Premature elevation of progesterone might disrupt the late follicular phase developmental microenvironment, impairing granulosa cell function and the final maturation of oocytes (37, 38), consequently compromising embryo quality. Handa et al. (39) further confirmed that the PPOS group had a lower rate of high-quality cleavage-stage embryos and a significantly lower LBR than the GnRH-ant group, with elevated mitochondrial DNA gene expression, suggesting potential impacts on embryo quality or endometrial receptivity. Although the PPOS protocol uses a freeze-all strategy to avoid decreased endometrial receptivity and inadequate luteal function associated with fresh embryo transfer under pituitary suppression, in women of advanced reproductive age, pituitary function recovery itself may have inherent limitations, and baseline luteal function may already be compromised (40, 41). This could also be an important contributing factor to the lower LBR in this group. Chen H et al. (42) suggested that within the subgroup of women with severely diminished ovarian reserve (AFC ≤ 5) and advanced age, the GnRH-ant protocol may be more likely to achieve live births compared to the PPOS protocol. Regarding miscarriage rates, while a statistically non-significant difference was observed between the two groups, the miscarriage rate in the PPOS group was lower than that in the GnRH-ant group, consistent with previous findings (43). This warrants further investigation. It is noteworthy that, in accordance with the Chinese consensus guidelines, miscarriage in this study was defined as spontaneous abortion occurring before 28 weeks of gestation or before a fetal weight of less than 1000 g, whereas the internationally recognized thresholds for miscarriage are typically 20 or 24 weeks of gestation. Therefore, the miscarriage rate reported in this study is slightly higher than if the international definitions had been applied.

The strength of this study lies in its focus on a specific patient population of women with ARA, employing PSM to control for intergroup baseline balance. After matching, the SMD of all covariates were < 0.2, effectively minimizing baseline bias and ensuring comparability between groups. Furthermore, this study used LBR as the primary outcome, the gold standard for evaluating the effectiveness of ART procedures, providing greater clinical relevance for women with ARA and supporting the optimization of treatment strategies in this population.

This study has limitations inherent to its retrospective design and single-center setting. Additionally, the balance of ovarian reserve parameters, such as AFC and AMH, was suboptimal, as indicated by the SMD analysis for confounding factors. Sun X et al. (44) reported that AFC and AMH are positively correlated with the number of oocytes retrieved. Therefore, the difference in AFC and AMH may potentially contribute to subsequent embryological and pregnancy outcomes. However, subgroup analyses of AFC and AMH are limited by the sample size, which may possibly mask interactions between protocol efficacy and patient characteristics. Although we effectively controlled for measured baseline biases through PSM, the possibility of unmeasured confounders, such as the exact duration of infertility and etiologies of infertility, or socioeconomic factors, cannot be excluded, and these factors may potentially influence the study results. Given these limitations, future studies should focus on prospective, large-sample randomized controlled trials with specified subgroup analyses for comparing these two treatment protocols across diverse ARA female subpopulations.

This study indicates that for infertile women of ARA age undergoing frozen embryo transfer, the GnRH-ant protocol appeared superior to PPOS protocol, as evidenced by improved embryological outcomes and higher clinical pregnancy rates, with LBRs exhibiting a similar trend. Its advantages include higher ovarian response modulation, a higher yield of utilizable embryos, and a significantly improved LBR. For patients of ARA age, the GnRH-ant protocol may provide a relatively greater prospect of achieving live birth and merits priority consideration.

## Data Availability

The raw data supporting the conclusions of this article will be made available by the authors, without undue reservation.
